# cBIN1 Score (CS) Identifies Ambulatory HFrEF Patients and Predicts Cardiovascular Events

**DOI:** 10.3389/fphys.2020.00503

**Published:** 2020-05-25

**Authors:** Tara C. Hitzeman, Yu Xie, Ronit H. Zadikany, Andriana P. Nikolova, Rachel Baum, Ana-Maria Caldaruse, Sosse Agvanian, Gil Y. Melmed, Dermot P. B. McGovern, Dael R. Geft, David H. Chang, Jaime D. Moriguchi, Antoine Hage, Babak Azarbal, Lawrence S. Czer, Michelle M. Kittleson, Jignesh K. Patel, Alan H. B. Wu, Jon A. Kobashigawa, Michele Hamilton, TingTing Hong, Robin M. Shaw

**Affiliations:** ^1^Nora Eccles Harrison Cardiovascular Research and Training Institute, University of Utah, Salt Lake City, UT, United States; ^2^Department of Medicine, Cedars-Sinai Medical Center, Cedars-Sinai Smidt Heart Institute, Los Angeles, CA, United States; ^3^Division of Gastroenterology, Department of Medicine, Cedars-Sinai Medical Center, Los Angeles, CA, United States; ^4^Department of Laboratory Medicine, University of California, San Francisco, San Francisco, CA, United States

**Keywords:** heart failure, cardiac muscle remodeling, ion channels, calcium handling, cBIN1 score

## Abstract

**Background:**

Cardiac Bridging Integrator 1 (cBIN1) is a membrane deformation protein that generates calcium microdomains at cardiomyocyte t-tubules, whose transcription is reduced in heart failure, and is released into blood. cBIN1 score (CS), an inverse index of plasma cBIN1, measures cellular myocardial remodeling. In patients with heart failure with preserved ejection fraction (HFpEF), CS diagnoses ambulatory heart failure and prognosticates hospitalization. The performance of CS has not been tested in patients with heart failure with reduced ejection fraction (HFrEF).

**Methods and Results:**

CS was determined from plasma of patients recruited in a prospective study. Two comparative cohorts consisted of 158 ambulatory HFrEF patients (left ventricular ejection fraction (LVEF) ≤ 40%, 57 ± 10 years, 80% men) and 115 age and sex matched volunteers with no known history of HF. N-terminal pro-B-type natriuretic peptide (NT-proBNP) concentrations were also analyzed for comparison. CS follows a normal distribution with a median of 0 in the controls, which increases to a median of 1.9 (*p* < 0.0001) in HFrEF patients. CS correlates with clinically assessed New York Heart Association Class (*p* = 0.007). During 1-year follow-up, a high CS (≥ 1.9) in patients predicts increased cardiovascular events (43% vs. 26%, *p* = 0.01, hazard ratio 1.9). Compared to a model with demographics, clinical risk factors, and NT-proBNP, adding CS to the model improved the overall continuous net reclassification improvement (NRI 0.64; 95% CI 0.18-1.10; *p* = 0.006). Although performance for diagnosis and prognosis was similar to CS, NT-proBNP did not prognosticate between patients whose NT-proBNP values were > 400 pg/ml.

**Conclusion:**

CS, which is mechanistically distinct from NT-proBNP, successfully differentiates myocardial health between patients with HFrEF and matched controls. A high CS reflects advanced NYHA stage, pathologic cardiac muscle remodeling, and predicts 1-year risk of cardiovascular events in ambulatory HFrEF patients. CS is a marker of myocardial remodeling in HFrEF patients, independent of volume status.

## Introduction

Heart failure (HF) is currently one of the most burdensome global public health problems. This complex, progressive condition is caused by a cascade of pathophysiologic insults that lead to abnormally functioning myocardium, which at the myocyte level is associated with disturbed intracellular calcium homeostasis and impaired mitochondrial function and cellular metabolism. The current prevalence of HF among adults in the United States is 6.2 million and is projected to keep increasing over the next 10 years (Virani et al., 2020). Half of this population have reduced ejection fraction (HFrEF) with high morbidity and mortality mainly caused by cardiac pump failure and sudden cardiac death. Clinical management remains challenging, with a total cost of care greater than $30 billion per year. HF hospitalizations are already the highest single cost to Medicare for Americans over the age of 65, and this expense is expected to increase (Go et al., 2014). The substantial burden of this disease represents a healthcare and economic imperative to assess the health of cardiac muscle and use that information to limit future hospitalizations.

Unlike with most cancers which have advanced tissue based molecular diagnostics, our current methods at assessing prognosis in heart failure are more limited to *status quo* hemodynamics and organ function assessments (Murray et al., 2005; Chow and Senderovich, 2018; Zadikany et al., 2019). Current guidelines define HFrEF patients as having transthoracic echocardiogram obtained left ventricular ejection fraction (LVEF) of less than 40% (Yancy et al., 2013). Traditional diagnostic tools for assessing the severity of HFrEF include not only the transthoracic echocardiogram (Loeppky et al., 1984), but New York Heart Association (NYHA) assessment (Dickstein et al., 2008), cardiopulmonary exercise testing (Weber and Janicki, 1985; Corra et al., 2002), and cardiac index obtained from right heart catheterization (Ganz et al., 1971). These assessments provide valuable information regarding overall cardiac function (Ponikowski et al., 2016). However, because these assessments will vary with loading conditions, they do not necessarily correlate with intrinsic myocardial health. Furthermore, current cardiac assessment tools often require specialized equipment and highly trained medical staff, limiting accessibility for patients being seen in a general clinician’s office. Clinicians could be well served with a quantitative blood-based tool that is able to effectively assess the molecular health and reserve of cardiac muscle to assist with critical clinical decision making such as the need for left ventricular assist device (LVAD) and implantable cardioverter defibrillator (ICD), as well as heart transplant priorities.

The gold standard biomarkers to assess patients with HF symptoms are the brain natriuretic peptide (BNP) family (Sun et al., 2014). Both BNP and its more stable and non-active version, N-terminal prohormone BNP (NT-proBNP) are secreted by cardiomyocytes in response to pressure and stretch. Active BNP results in a downstream natriuresis, diuresis, and vasodilatation (Lee and Daniels, 2016). As stretch-response molecules, blood available BNP biomarkers help assess fluid status and assist in evaluating patients with acute dyspnea (Januzzi et al., 2005). However, NT-proBNP is not more effective than a usual care strategy in high-risk patients with HFrEF (Felker et al., 2017). Furthermore, BNP values are affected by renal function and body mass index (BMI) (Myhre et al., 2018). Taken together, HF diagnosis and management of ambulatory patients could benefit from a quantitative assessment of the health of cardiac muscle that is insensitive to volume, BMI, or non-cardiac organ function.

Cardiac bridging integrator 1 (cBIN1) score (CS), a novel biomarker of HF, has recently been introduced as a diagnostic and prognostic test in patients with heart failure with preserved ejection fraction (HFpEF) (Nikolova et al., 2018). cBIN1 is a cardiac-specific transverse tubule (t-tubule) membrane sculpting protein, functioning to organize microdomains responsible for calcium release and excitation-contraction (EC) coupling (Hong et al., 2014), as well as efficient diastolic calcium reuptake (Liu et al., in press). In doing so, cBIN1 helps maintain intracellular calcium homeostasis particularly at functionally important microenvironment adjoining t-tubule, junctional sarcoplasmic reticulum, and mitochondria. As a result, low cardiomyocyte cBIN1, which is also measurable at the plasma level (Xu et al., 2017), is intrinsically linked to cardiac inotropy (Hong et al., 2012), lusitropy (Liu et al., in press), and arrhythmia risks (Hong et al., 2014) particularly during stress response (Fu et al., 2016). Derived from the inverse of plasma cBIN1 level (Nikolova et al., 2018), CS rises with worsening HF. In this study, we explored whether CS has the potential to diagnose HFrEF when compared with matched controls. We also explored CS’s ability, within the HFrEF cohort, to accurately predict future cardiac events.

## Materials and Methods

### Study Design

All human studies were approved by the institutional review board at Cedars-Sinai Medical Center. Full informed consent was obtained from all subjects prior to participation in the study. The study involved two human populations, including patients with documented HFrEF and volunteers with no known history of HF.

The HFrEF cohort, followed longitudinally in the Advanced Heart Failure clinic at Cedars-Sinai Smidt Heart Institute, consisted of those in the clinic with a known diagnosis of HFrEF (left ventricular ejection fraction (LVEF) ≤ 40% and history of HF). From July 2014–November 2015, 158 patients were enrolled and a blood sample was obtained from patients at the time of clinic-scheduled phlebotomy. Patients with LVEF > 40% at the time of enrollment were excluded. Patient demographics, clinical information, medications, and laboratory and diagnostic test results were gathered from the hospital electronic medical records into a secure de-identified database. Subsequent clinical information was updated from chart review occurring every 3 months for 1 year.

The comparison cohort, consisting of 115 age and sex matched volunteers with no known history of HF, was obtained from the Cedars-Sinai MIRIAD IBD Consortium and Innovative Research, under similar plasma collection and preparation as the HFrEF samples. A clinical history, including patient demographics, medical history, and current medications was obtained from each volunteer.

### Sample Processing and CS Determination

Whole venous blood was drawn into EDTA lavender tubes, stored immediately at 4°C for less than 4 h, and then processed to plasma and flash frozen for storage at −80°C prior to use, as previously described (Nikolova et al., 2018). The concentration of cBIN1 was determined using a cBIN1 specific sandwich-ELISA assay provided by Sarcotein Diagnostics, as previously described (Xu et al., 2017). Findings are reported using CS, the natural log of the inverse of cBIN1 plasma concentration (Nikolova et al., 2018), which increases as cBIN1 decreases.

### NT-proBNP Assay

NT-proBNP values were obtained from the plasma of control and HFrEF patients. The Cedars-Sinai Medical Center clinical laboratory referred samples to Quest Diagnostics Laboratory to perform the NT-proBNP assay using electrochemiluminescence.

### Cardiac Event Defined as the Primary Outcome During Follow-Up

Cardiac events were predefined as any HF or cardiac hospitalization, left ventricular assist device (LVAD) or mechanical circulatory support (MCS) placement, orthotopic heart transplantation (OHT), or death within the 1 year after time of blood draw. All chart review and adjudication were done by a two-physician panel, who were not involved in the clinical care of the patients (YX and RZ).

### Statistical Analysis

Data distributions were assessed for normality by the Kolmogorov-Smirnov test. Continuous variables with normal distributions were expressed as means and standard deviations and compared using two-sided *t*-tests. Continuous variables with non-normal distributions were analyzed with medians and interquartile ranges (IQR) and compared using Mann–Whitney U and Kruskal–Wallis tests. Categorical variables were compared using Fisher’s exact or chi-square test. Receiver operating characteristic (ROC) analyses were performed to determine the sensitivity and specificity of CS and NT-proBNP to diagnose HF. Kaplan-Meier and Cox-proportional hazard analyses were used to compare the differences in event-free survival rates between patients with high and low values of CS. Model one included age, sex, BMI, NYHA class, LVEF, estimated glomular filtration rate (eGFR), and NT-proBNP. Model two included the same covariants with CS added to it. To determine the increase in discriminative value to predict 1 year outcomes following the addition of CS as a covariant in the survival model, we evaluated continuous net reclassification improvement (NRI). Two-sided *p*-values were reported and a *p* < 0.05 was considered statistically significant. Statistical analyses were conducted using SAS Version 9.3.1 software (SAS Institute, Inc.), RStudio Version 1.0.143 (RStudio, Inc.), and GraphPad Prism Version 6 (GraphPad Software).

## Results

### Study Cohorts

158 HFrEF patients (57 ± 10 years old, 80% men) were age and sex matched to 115 volunteers with no known history of HF (54 ± 6 years old, 80% men) (Table 1). The HFrEF cohort consisted of 55 (35%) ischemic and 101 (64%) non-ischemic patients. Most of the non-ischemic patients have idiopathic cardiomyopathy (68%), with the remainder due to valvular disease, toxin-mediated disease, or infiltrative disease. Most patients were classified as New York Heart Association (NYHA) II or III (35 and 48%, respectively) and the prevalence of comorbidities was 45% hypertension, 37% diabetes, and 23% chronic kidney disease. The baseline median LVEF on transthoracic echocardiography was 24 ± 8%. Patients were treated with guideline-directed medical therapy by the Cedars-Sinai Advanced Heart Disease group.

**TABLE 1:** Baseline characteristics of HFrEF and matched controls.

Characteristics (%)	Heart failure with reduced EF (*n* = 158)	Matched controls (*n* = 115)	*p*-value
Age (SD)	57 ± 10.4	54 ± 6.1	<0.001
≤ 50 years	39 (25)	29 (25)	NS
> 50 years	119 (75)	86 (75)	
Male	126 (80)	92 (80)	NS
White	93 (59)	67 (58)	NS
BMI, kg/m2 (SD)	29 ± 6.1	29 ± 5.6	NS
Hypertension	71 (45)	13 (11)	<0.001
Diabetes	59 (37)	8 (7)	<0.001
CKD	36 (23)		
LVEF	24 ± 8.1		
**Medications**			
Beta-Blockers	139 (88)		
ACE-I/ARB	120 (76)		
Diuretics	125 (79)		
**NYHA**			
I	21 (13)		
II	55 (35)		
III	76 (48)		
IV	6 (4)		
**Subtype of heart failure**			
Ischemic	55 (35)		
Non-Ischemic	101 (64)		
Valvular	10 (10)		
Dilated	11 (11)		
Toxin	9 (9)		
Infiltrative	2 (2)		
Other	69 (68)		

*HFrEF, heart failure with reduced ejection fraction; SD, standard deviation; BMI, body mass index; CKD, chronic kidney disease; LVEF, left ventricular ejection fraction; ACE-I, angiotensin converting enzyme inhibitor; ARB, angiotensin receptor blocker; NYHA, New York Heart Association.*

### CS Is Elevated in Patients With HFrEF

**FIGURE 1:**
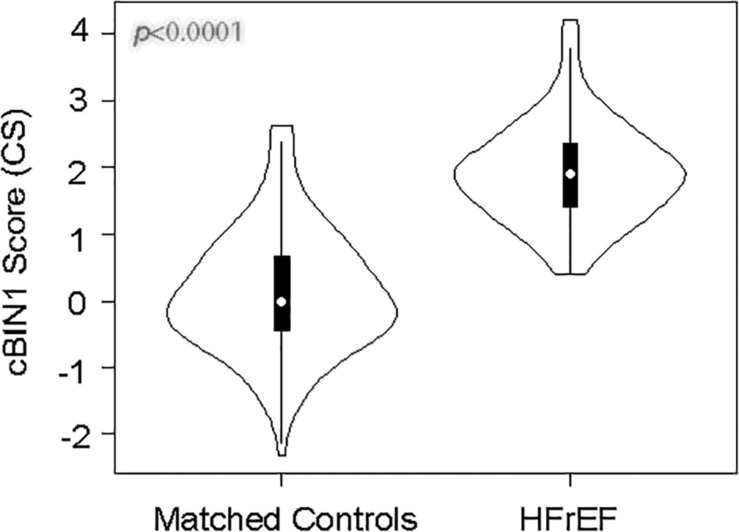
Distribution of CS among HFrEF and matched controls. The vertical axis is the cBIN1 score (CS), and the width of each violin graph depicts the density plot at each measured value. The HFrEF cohort median CS is elevated relative to the controls, 1.9 (IQR 1.4–2.4) compared to 0 (IQR -0.5–0.7), respectively (*p* < 0.0001).

Violin plots of CS in HFrEF and matched controls are shown in Figure 1. The HFrEF cohort median CS is elevated relative to the controls, 1.9 (IQR 1.4–2.4; mean ± SD 1.9 ± 0.7) compared to 0 (IQR -0.5–0.7; mean ± SD 0.1 ± 0.9), respectively (*p* < 0.0001), with an approximately normal distribution in both cohorts. The HFrEF cohort median of ln NT-proBNP level, 7.0 (IQR 6.0–8.1), is also significantly elevated compared to the median level of the control cohort, 3.3 (IQR 2.7–4.1), with *p* < 0.0001 (Supplementary Figure S1). NT-proBNP levels (pg/ml) have a median of 28 (IQR 15–60) in controls and a median of 1,081 (IQR 409–3,419) in HFrEF patients.

Median CS (Table 2) and NT-proBNP (Supplementary Table S1) values were analyzed among different subgroups of demographics and clinical parameters. CS and NT-proBNP do not vary with sex or age. Unlike NT-proBNP, CS does not differ among BMI categories (normal, overweight, and obese) in controls or HFrEF patients. Among HFrEF patients with normal vs. decreased estimated glomerular filtration rate (eGFR < 60 ml/min/m^2^), CS does not differ (1.8 and 1.9, respectively), while patients with worsening eGFR had increased NT-proBNP levels (*p* = 0.01). CS an NT-proBNP correlate with clinically assessed New York Heart Association Class (*p* = 0.007 and *p* < 0.001, respectively). The lack of association with CS and obesity or renal function is consistent with results previously reported in patients with HFpEF and matched controls (Nikolova et al., 2018).

**TABLE 2:** CS among subgroups in matched controls and HFrEF patients.

Characteristics	*N*	Matched controls	IQR (Q1-Q3)	*p*-value	N	HFrEF	IQR (Q1-Q3)	*p*-value
**All patients**	115	0.0	−0.5–0.7	–	158	1.9	1.4–2.4	–
**Sex**				NS				NS
Men	92	0.0	−0.5–0.8		126	1.9	1.4–2.3	
Women	23	0.0	−0.3–0.4		32	1.9	1.3–2.4	
**Age (years)**				NS				NS
< 55	67	−0.1	−0.5–0.7		54	1.8	1.5–2.1	
≥ 55	48	0.1	−0.4–0.7		104	2.0	1.4–2.4	
**Race/Ethnicity**				NS				0.03
White	67	0.1	−0.6–0.8		93	1.8	1.3–2.1	
Black	24	0.0	−0.5–0.9		26	2.1	1.8–2.6	
Hispanic	24	−0.1	−0.3–0.4		28	2.0	1.5–2.5	
Asian	0	–	–		9	1.6	1.3–2.3	
**BMI** (kg/m^2^)				NS				NS
Normal (<25)	32	0.0	−0.5–0.7		29	1.9	1.4–2.4	
Overweight (25–29.9)	34	0.0	−0.4–0.7		65	1.9	1.4–2.4	
Obese (30–34.9)	25	−0.1	−1.0–0.4		38	2.0	1.7–2.5	
Morbid Obesity (≥35)	14	−0.2	−0.4–0.1		25	1.7	1.1–2.1	
**Etiology**								NS
Ischemic HFrEF					55	2.0	1.5–2.5	
Non-ischemic HFrEF					101	1.9	1.4–2.3	
**eGFR (ml/min/m^2^)**								NS
<60					94	1.9	1.5–2.2	
>60					64	1.8	1.4–2.5	
**NYHA**								0.007
I					21	1.6	1.3–2.0	
II					55	1.8	1.5–2.3	
III					76	2.0	1.4–2.5	
IV					6	2.6	2.1–3.0	
**OHT**								0.0006
No					127	1.8	1.4–2.2	
Yes					31	2.3	1.7–3.0	

*HFrEF, heart failure with reduced ejection fraction; IQR, interquartile range; Q1, quartile 1; Q3, quartile 3; BMI, body mass index; eGFR, estimated glomular filtration rate; NYHA, New York Heart Association; OHT, orthotopic heart transplant.*

### CS Diagnoses Failing Heart Muscle

**FIGURE 2:**
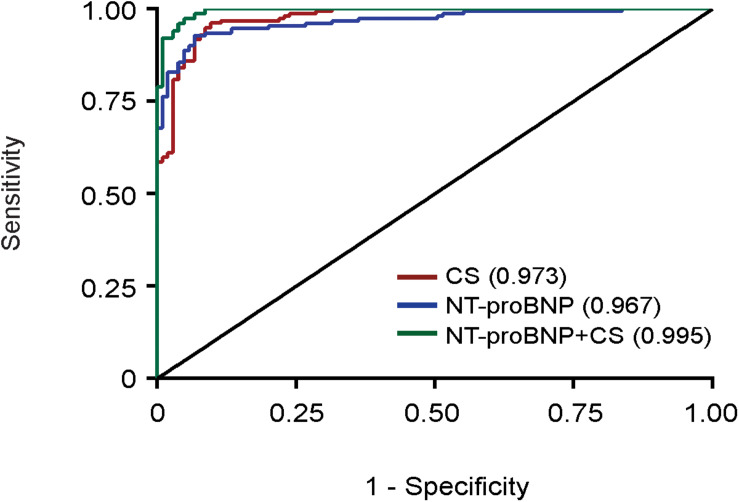
Receiver operating characteristic (ROC) curves. ROC curve containing the various sensitivities and specificities of CS (AUC = 0.973, red), NT-proBNP (AUC = 0.967, blue), and combined NT-proBNP and CS (AUC = 0.995, green) to diagnose disease in our control and HFrEF cohorts.

Since CS is higher in HFrEF patients than controls, we generated receiver operating characteristic (ROC) curves for CS and NT-proBNP, adjusting for age, sex, and BMI (Figure 2). The area under the curve (AUC) of the ROC indicates that both CS (AUC = 0.973, red) and NT-proBNP (AUC = 0.967, blue) distinguish patients with HF from the control population. When CS and NT-proBNP were combined, the ability to determine HF from the control population, while already strong, still improved with statistical significance (AUC = 0.995, green, *p* = 0.01) suggesting that CS (muscle health) and NT-proBNP (intracardiac volume) can be complementary.

### Elevated CS Prognosticates Cardiovascular Hospitalization in HFrEF Patients

**FIGURE 3:**
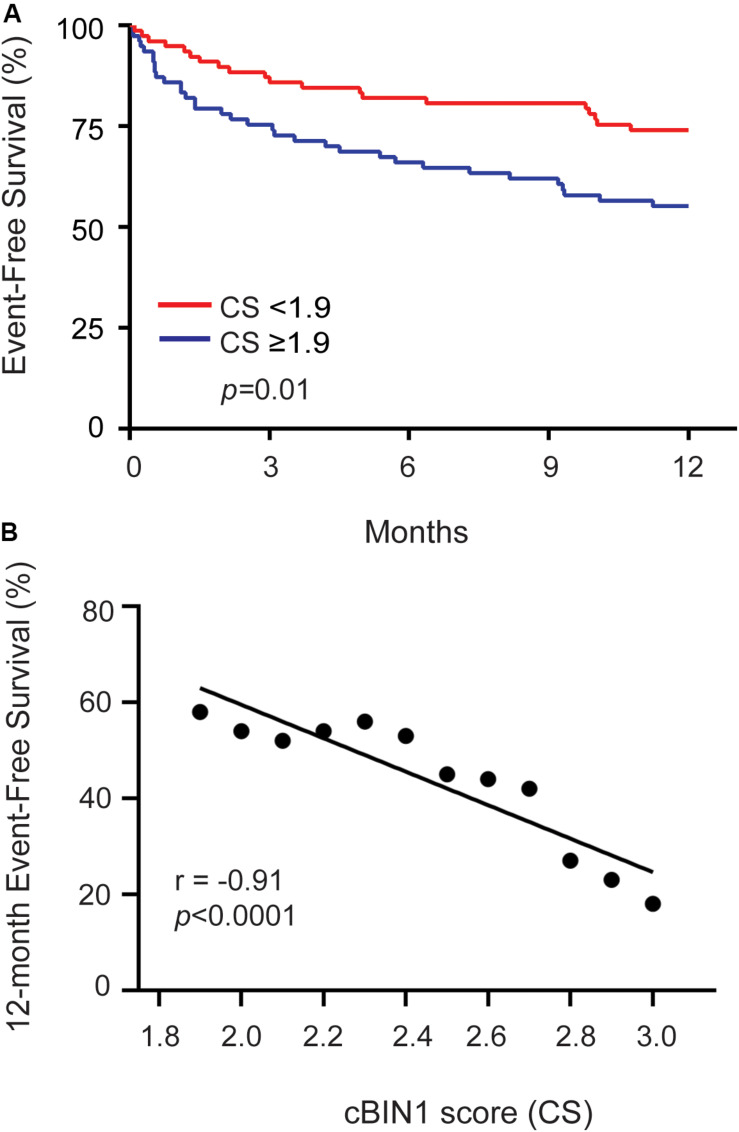
Cardiovascular event free survival of HFrEF patients during 12-month follow-up. **(A)** Kaplain-Meier survival curve is shown here for all HFrEF patients. The red line demonstrates patients with CS < 1.9 and the blue line demonstrate patients with CS ≥ 1.9. Event free survival is defined as patients who did not have a cardiovascular (CV) event (HF-related hospitalization, cardiac hospitalization, LVAD, OHT, or death) during 12-months follow-up. A low CS (<1.9) predicted a higher event-free survival among all HFrEF patients (*p* = 0.01). **(B)** Scatterplot of 12-month event-free survival vs. CS for all CS ≥ 1.9 indicates a negative correlation (Pearsons’s correlation coefficient −0.91, *p* < 0.0001).

Next, we explored whether, in addition to its diagnostic value, CS can serve as a prognostic marker in predicting future clinical outcomes in patients with HFrEF. During the 12-month follow-up period, we found that 66 patients (42%) had at least one cardiovascular (CV) event: 41 (26%) patients had a HF-related hospitalization, 16 (10%) patients with a hospitalization diagnosis that was cardiac, but not HF, in origin, 31 (20%) patients had OHT, 4 (3%) went onto MCS or LVAD, and 9 (6%) patients died (6 of which were due to cardiac etiology). Kaplan-Meier survival curves were generated (Figure 3A). The median CS level of 1.9 (drawn at the initial visit) was used as a cutoff to differentiate high verse low CS (Nikolova et al., 2018). A cutoff higher than the median of 1.9 further increases specificity of 12-month event free survival (Figure 3B). Using a model including age, sex, BMI, NYHA class, LVEF, eGFR, and NT-proBNP (Table 3), the hazard ratio (HR) of a high CS ≥ 1.9 predicting CV event rate among HFrEF patients during 1 year of follow-up was 1.9 (43 vs. 26%, *p* = 0.03). The addition of CS ≥ 1.9 significantly improved continuous net reclassification improvement (NRI 0.64; 95% CI 0.18-1.10; *p* = 0.006).

**TABLE 3:** Risk of CV Event in HFrEF patients with multivariate Cox regression analysis.

Variables	Model without CS			Model with CS		
	HR	95% CI	*p*-value	HR	95% CI	*p*-value
Age, > 55 vs. < 55 years	0.88	0.45–1.72	0.71	0.95	0.49–1.86	0.88
Sex, female vs. male	0.45	0.20–1.03	0.06	0.47	0.20–1.07	0.07
BMI, kg/m^2^	1.0	0.98–1.09	0.25	1.04	0.98–1.1	0.19
NYHA class, III-IV vs. I-II	2.75	1.39–5.43	<0.01	2.59	1.33–5.06	0.01
LVEF%	1.03	0.99–1.07	0.14	1.04	1.00–1.08	0.07
eGFR, ml/min/m^2^	1.00	0.98–1.01	0.53	0.99	0.98–1.01	0.36
NT-proBNP, Q4 vs. Q1	3.05	1.00–9.30	0.05	3.30	1.1–10.0	0.03
CS, >1.9 vs. <1.9	–	–	–	1.93	1.07–3.48	0.03

*CV, cardiovascular; HFrEF, heart failure with reduced ejection fraction; HR, hazard ratio; CI, confidence interval; eGFR, estimated glomular filtration rate; BMI, body mass index; NYHA, New York Heart Association; LVEF, left ventricular ejection fraction.*

For NT-proBNP, the Kaplan-Meier survival curve using the median NT-proBNP cutoff value of 1078 pg/ml, was not quite significant in predicting 1-year CV event (40 vs. 28%, *p* = 0.06). Interestingly, the prognostic power of NT-proBNP was in the low values (<409 pg/ml in the 1st quartile) (Supplementary Figure S2). A low NT-proBNP predicted CV events with a HR of 3.3 (40 vs. 19%, *p* = 0.03). There was no significant prognostication in patients with NT-proBNP greater than 409 pg/ml.

## Discussion

The prevalence of HFrEF is increasing over time as our population continues to age, making this clinical syndrome a significant public health concern. The diagnosis of HF remains complex, involving a combination of history taking, physical examination, labs, imaging, and functional studies. Among the existing armamentarium of HF diagnostics, there is currently no invasive or non-invasive tool to measure intrinsic cardiomyocyte remodeling. CS can be used as a blood available biomarker to assist in this clinical need.

In pre-clinical animal models, pathophysiological changes in cardiomyocytes of failing hearts such as t-tubule remodeling is considered the transition point from functional compensation to decompensated heart failure (Wei et al., 2010). cBIN1 reductions are associated with t-tubule remodeling and the progression of heart failure (Hong et al., 2012, 2014; Xu et al., 2017). With recovery of muscle, cBIN1 levels also recover (Lyon et al., 2012). Given that cBIN1 is also blood available, CS provides a blood available “liquid biopsy” of cardiac muscle.

HF progression can be reflected by different biomarkers, with robust data demonstrating the ability of natriuretic peptides (BNP and NT-proBNP) to reflect myocardial wall stress (Iwanaga et al., 2006). As a marker of acute volume overload, the diagnostic utility of BNP peptides is well-validated in detecting acute decompensated HF (Januzzi et al., 2005). However, blood BNP values are affected by obesity and renal dysfunction, in addition to requiring adjustments for age and sex (Myhre et al., 2018). Furthermore, because natriuretic peptides are reflective of volume status, it can be difficult to use BNP as a marker to distinguish between patients with severe HFrEF and patients with non-cardiac origin volume overload, such as renal failure.

There is a need for additional HF biomarkers that can help identify the intrinsic health of cardiac muscle cells independent of volume status. Because CS reflects cardiac muscle cell health, it is stable and independent of fluctuations induced by intracardiac volume, inflammatory state, or body habitus (Nikolova et al., 2018). The characteristic performance of CS observed in the current HFrEF cohort indicates that a positive CS can help accurately identify failing heart muscle and is not sensitive to comorbid conditions. Thus, CS provides an unprecedent and non-invasive tool to help with evaluation of myocyte health from EC-coupling efficiency to electrical stability, determining individual’s risks of pump failure and arrhythmias. As a signature footprint from myocytes, CS is therefore particularly important at many critical clinical decision-making points for HFrEF patients, guiding medical treatment choices, clinical surveillance intervals, criteria determination for LVAD and ICD implant, and evaluation of the need and the recovery potential of a heart transplant.

In this report we find that within a HFrEF cohort, a CS cutoff value of greater than 1.9 accurately predicts cardiac hospitalization during a 1 year follow-up period (Figure 3), as it did in a HFpEF cohort (Nikolova et al., 2018). In addition, our findings indicate that the addition of CS, to a model with established risk predictors, significantly improved risk classification for CV events in HFrEF patients. In an ambulatory clinic, a high CS may provide the added prognostic information needed to help sway the pendulum of clinical care toward more aggressive surveillance or escalating care to more advanced therapies. Conversely, an advanced HFrEF patient with a low, even normal CS, may indicate an ability to postpone advanced therapies and continue monitoring with periodic clinic visits. Clinical decompensation with a high CS would indicate failing heart, whereas clinical decompensation with a low CS could suggest extracardiac factors such as renal failure or medical non-compliance are dominating the clinical decline.

### Limitations

This is a real-world single center clinical patient population with no exclusion criteria and the HFrEF patients had follow up at the discretion of clinical providers. We would like to reproduce these findings in a prospective multicenter HFrEF cohort. In keeping with our prior study, we used a CS cutoff of the median (1.9). Based on the data in Figure 3B, investigators may choose to use a higher cutoff than the median CS, which would improve test specificity.

## Conclusion

A protein involved in cardiomyocyte t-tubule remodeling is blood available and can function as a biomarker that helps diagnose and prognosticate ambulatory HFrEF patients. The potential advantages of CS include early pre-clinical diagnosis in asymptomatic patients, differentiating cardiac origin volume overload from extracardiac source, prognosticating outcomes in stable ambulatory patients, and evaluating myocardial recovery once on a successful therapeutic regimen. In an era of ballooning health care costs, CS is a blood-available test that substantially adds to the assessment of ambulatory HFrEF.

## Data Availability Statement

The data that support the findings of this study are available from the corresponding author, RMS, upon reasonable request.

## Ethics Statement

The studies involving human participants were reviewed and approved by Cedars-Sinai Institutional Review Board. The patients/participants provided their written informed consent to participate in this study.

## Author Contributions

TCH, TTH, and RS contributed to conception and design of the study. TCH, YX, and RZ organized the database. TCH performed the statistical analysis. YX wrote the first draft of the manuscript. TCH, YX, RZ, AN, MK, MH, AW, TTH, and RS wrote sections of the manuscript. GM, DM, DG, DC, JM, AH, BA, LC, MK, JP, JK, and MH consented patients and collected patient samples. RB, A-MC, and SA ran ELISA assays.

## Conflict of Interest

The authors declare that the research was conducted in the absence of any commercial or financial relationships that could be construed as a potential conflict of interest.
