# Supplementary material for: cBIN1 Score (CS) Identifies Ambulatory HFrEF Patients and Predicts Cardiovascular Events
Source: Front Physiol. 2020 May 25;11:503. doi: 10.3389/fphys.2020.00503 (PMC7326053; doi:10.3389/fphys.2020.00503)
Supplement: Supplementary file 1 [file Data_Sheet_1.PDF]

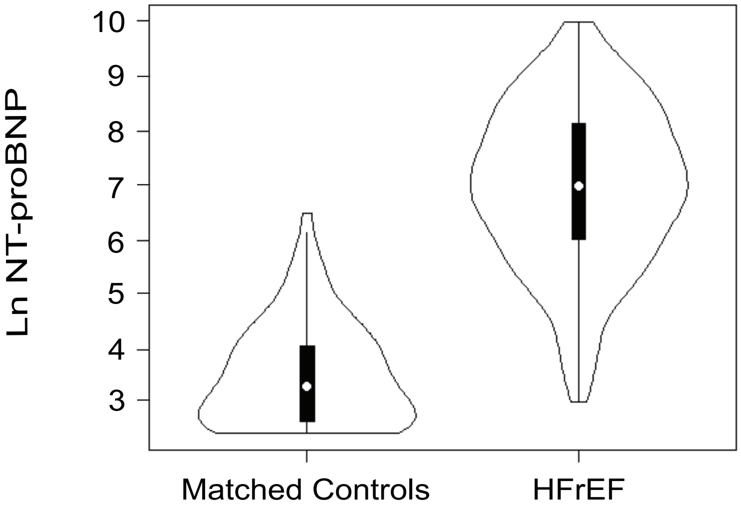

Supplemental Figure 1. Distribution of NT-proBNP among HFrEF and matched controls. The HFrEF cohort median of ln NT-proBNP levels, 7.1 (IQR 5.9–8.2) is significantly elevated compared to the median level of the control cohort, 3.3 (IQR 2.7–4.1), with $p<0.0001$.

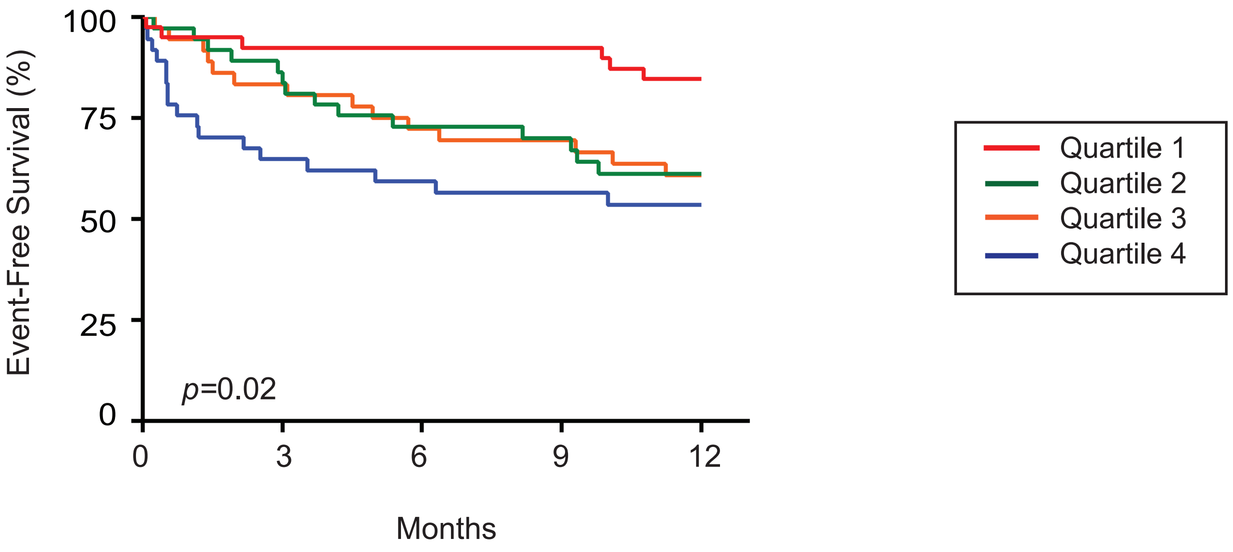

Supplemental Figure 2. Kaplan-Meier of HFrEF patients who are free of cardiovascular event during 12-months follow-up. Kaplain-Meier survival curve is shown here for all HFrEF patients divided into the quartiles of NT-proBNP. The red line demonstrates patients in quartile 1, the green line demonstrates patients in quartile 2, the orange line demonstrates patients in quartile 3, and the blue line demonstrate patients in quartile 4. Event free survival is defined as patients who did not have a cardiovascular (CV) event (HF-related hospitalization, cardiac hospitalization, LVAD, OHT, or death) during 12-months follow-up. A high NT-proBNP in the 4th quartile (>3,419 pg/ml) predicted a higher event-free survival among all HFrEF patients ($p=0.02$).

Supplemental Table 1. NT-proBNP among subgroups in matched controls and HFrEF patients

Characterisitcs	N	Matched Controls	IQR (Q1-Q3)	*p*-value	N	HFrEF	IQR (Q1-Q3)	*p*-value
**All patients**	115	28	15 - 60	----	153	1081	409 - 3419	----
**Sex**				NS				NS
Men	92	27	14 - 58		31	1165	409 - 3529	
Women	23	29	18 - 118		122	778	230 - 2078	
**Age (years)**				NS				NS
< 55	67	29	15 - 65		53	1013	381 - 2983	
≥ 55	48	27	16 - 58		100	1141	420 - 3486	
**Race/Ethnicity**				0.01				NS
White	67	33	19 - 75		90	905	370 - 2770	
Black	24	30	13 - 66		26	1675	233 - 5842	
Hispanic	24	14	12 - 41		28	1588	591 - 4867	
Asian	0	----	----		8	964	467 - 1357	
**BMI** (kg/m^2)				<0.001				NS
Normal (< 25)	32	58	23 - 95		27	1714	454 - 4412	
Overweight (25-29.9)	34	28	15 - 47		63	1081	475 - 4321	
Obese (30-34.9)	25	28	14 - 58		37	1108	525 - 2983	
Morbid Obesity (≥ 35)	14	14	12 - 19		25	443	257 - 1153	
**Etiology**								NS
Ischemic HFrEF					55	1081	349 - 3424	
Non-ischemic HFrEF					96	1081	449 - 3371	
**eGFR (ml/min/m^2)**								0.01
< 60					61	1664	685 - 5193	
> 60					92	904	311 - 2107	
**NYHA**								<0.0001
I					19	315	183 - 475	
II					55	962	271 - 1787	
III					74	1730	815 - 4412	
IV					5	3628	1805 - 5467	
**OHT**								
No					125	1013	315 - 3008	0.02
Yes					28	1595	845 - 4991	

HFrEF = heart failure with reduced ejection fraction; IQR = interquartile range; Q1 = quartile 1; Q3 = quartile 3; BMI = body mass index; eGFR = estimated glomular filtration rate; NYHA = New York Heart Association; OHT = orthotopic heart transplant
